# Cardiac regenerative potential of cardiosphere-derived cells from adult dog hearts

**DOI:** 10.1111/jcmm.12585

**Published:** 2015-04-09

**Authors:** Michael Taylor Hensley, James de Andrade, Bruce Keene, Kathryn Meurs, Junnan Tang, Zegen Wang, Thomas G Caranasos, Jorge Piedrahita, Tao-Sheng Li, Ke Cheng

**Affiliations:** a Department of Molecular Biomedical Sciences, College of Veterinary Medicine, North Carolina State UniversityRaleigh, NC, USA; b Department of Clinical Sciences, College of Veterinary Medicine, North Carolina State UniversityRaleigh, NC, USA; c Center for Comparative Medicine and Translational Researches, North Carolina State UniversityRaleigh, NC, USA; d Department of Cardiology, First Affiliated Hospital, College of Medicine, Zhengzhou UniversityZhengzhou, China; e The Cyrus Tang Hematology Center, Soochow UniversitySuzhou, China; f Division of Cardiothoracic Surgery, University of North Carolina at Chapel HillChapel Hill, NC, USA; g Department of Stem Cell Biology, Atomic Bomb Disease Institute, Nagasaki UniversityNagasaki, Japan; h Joint Department of Biomedical Engineering, University of North Carolina at Chapel Hill and North Carolina State UniversityChapel Hill, NC, USA

**Keywords:** cardiosphere-derived cells, dogs, dilated cardiomyopathy, stem cell therapy

## Abstract

The regenerative potential of cardiosphere-derived cells (CDCs) for ischaemic heart disease has been demonstrated in mice, rats, pigs and a recently completed clinical trial. The regenerative potential of CDCs from dog hearts has yet to be tested. Here, we show that canine CDCs can be produced from adult dog hearts. These cells display similar phenotypes in comparison to previously studied CDCs derived from rodents and human beings. Canine CDCs can differentiate into cardiomyocytes, smooth muscle cells and endothelial cells *in vitro*. In addition, conditioned media from canine CDCs promote angiogenesis but inhibit cardiomyocyte death. In a doxorubicin-induced mouse model of dilated cardiomyopathy (DCM), intravenous infusion of canine CDCs improves cardiac function and decreases cardiac fibrosis. Histology revealed that injected canine CDCs engraft in the mouse heart and increase capillary density. Out study demonstrates the regenerative potential of canine CDCs in a mouse model of DCM.

## Introduction

Numerous animal studies 1–12 and the first-in-human CADUCEUS trial 13,14 have demonstrated the regenerative potential of cardiosphere-derived cells (CDCs) in ischaemic cardiomyopathy. Like human beings, veterinary patients such as domestic dogs also suffer from heart diseases. For instance, the overall prevalence of dilated cardiomyopathy (DCM) in Doberman Pinschers is greater than 50% 15. Once heart failure occurs, only palliative and sympathetic treatments exist. It is unknown whether canine CDCs could be derived from adult dogs and whether they are as potent as their human and rodent counterparts in cardiac regeneration. On the other hand, there is a growing need to improve (large) animal models for cell-based regenerative medicine 16. Dogs with naturally occurring heart diseases may serves as animal models of human diseases. Here, we derived CDCs from the adult dog heart and tested their regenerative potency in various *in vitro* and *in vivo* assays.

## Materials and methods

### Derivation and culture of Canine CDCs

Canine CDCs were generated and expanded as described 17 from myocardial specimens of a healthy dog heart donor 10,13. This tissue was surgically collected from the right ventricle of a 2-year old canine. An approximately, 6 mm × 6 mm piece of myocardial tissue was separated and washed with PBS (Life Technologies, Carlsbad, CA, USA). The tissue sample was then cut into smaller biopsy-sized pieces, and washed three times with PBS, followed by enzymatic digestion at 37°C in 5 mg/ml collagenase IV solution (Sigma-Aldrich, St. Louis, MO, USA) for 5 min. Iscove’s Modified Dulbecco’s Media (IMDM; Life Technologies, Carlsbad, CA, USA) containing 20% foetal bovine serum (FBS; Corning) is then added to the sample to inactivate the collagenase. After that, the tissue samples were further minced into smaller tissue explants (∼0.5 × 0.5 mm) before plating. Approximately, 50 pieces of tissue explants were then placed onto a fibronectin-coated plate with approximately 1.5 cm between each explant and covered with 2 ml of IMDM with 20% FBS overnight to aid the attachment of tissue explants. The cultures were maintained in IMDM with 20% FBS and media change was performed every other day. In about 1 week, cells started to outgrow from the tissue explants. Once these outgrowth cells are about 70–80% confluent, they were harvested by 5–10 min. of incubation with TryPLE Select™ (Life Technologies). The cells were then seeded into an Ultra-Low-attachment flask (Corning) at a density of 100,000 cells/cm^2^ and cultured in IMDM with 10% FBS. Phase-bright canine cardiospheres (CSps) started to form in 3–7 days. Canine CSps were then collected from the low-attachment flasks and re-plated onto fibronectin-coated surface to produce adherent canine CDCs. Canine CDCs were cultured in IMDM with 20% FBS media and passaged every 3–5 days. We used Passage 2-3 CDCs for all *in vitro* and *in vivo* testing. Canine mesenchymal stem cells (MSCs) were derived from the femur bone marrow of the dog donor and used as the Control cells. Canine MSCs were maintained in the same aforementioned CDC culture media 11,12.

### Clonal growth assay

Canine CDCs were seeded into a 96 well plate at a density of 1 cells per 100 μl per well. Only wells containing one cell were used for the experiment. Clonal growth of canine CDCs were tracked with a phase-bright microscope during 1-week period of time.

### Flow cytometry analysis

Canine CDCs were characterized by flow cytometry as described 7,10. Flow cytometry was performed on canine CDCs using a FACScalibur and LSR II (BD Biosciences, San Jose, CA, USA) and analysed using FlowJo software (TreeStar, Ashland, OR, USA). Cells were incubated with antibodies against CD105 (ab156756; Abcam, Cambridge, England), CD90 (bd555595; BD Biosciences), CD45 (mca1042a488; AbD Serotec, Kidlington, United Kingdom), CD117 (c-kit; ab5631; Abcam) for 60 min. Isotype-identical antibodies served as negative controls.

### Immunocytochemistry analysis

In addition to flow cytometry analysis, canine CDCs were seeded onto fibronectin-coated chamber slides, after which the cells were fixed with 4% paraformaldehyde (PFA), blocked/permeabilized with Protein Block Solution (DAKO, Carpinteria, CA, USA) containing 1% saponin (Sigma-Aldrich), and then stained with anti-CD105 (ab156756; Abcam), anti-CD90 (mca1036g; AbD Serotec), anti-c-kit (ab5631; Abcam) and anti-CD45 (mca1042a488; AbD Serotec) antibodies. Fluorescein isothiocyanate (FITC)-secondary antibodies (Abcam) were used in conjunction with the aforementioned primary antibodies.

### *In vitro* differentiation assay

Both canine MSCs and CDCs were cultured in the following differentiation media for 12 days: (*i*) cardiomyocyte differentiation media: IMDM with 1% N2 (Cat no. 12440; Gibco, Carlsbad, CA, USA), and 100 ng/ml Heregulin-β1 (Cat no. 100-03; Peprotech, Rocky Hill, NJ, USA); (*ii*) smooth muscle differentiation media: IMDM with 10 ng/ml platelet-derived growth factor-beta (Cat no. 100-14B; Peprotech) and (*iii*) endothelial differentiation media: IMDM with 50 ng/mL VEGF (Cat no. 100-20A; Peprotech) 18. After the differentiation process, the cells were fixed with 4% PFA, blocked/permeabilized with Protein Block Solution (DAKO) containing 1% saponin (Sigma-Aldrich), and then stained with mouse anti- α-sarcomeric actin (α-SA) (Sigma-Aldrich), mouse anti-smooth muscle actin (Sigma-Aldrich) and rabbit anti-von Willebrand factor (Abcam) antibodies. FITC or Texas-Red secondary antibodies were obtained from Abcam as well. Cell nuclei were counter-stained with 4’,6-diamidino-2-phenylindole (DAPI).

### Paracrine assay

Canine CDCs or MSCs were plated into a six-well plate and incubated in plain (serum-free) IMDM at a density of 1 million cell per 1 ml per well for 3 days. After that, the conditioned media (CM) from CDCs was harvested. Neonatal rat cardiomyocytes (NRCMs) were derived as described 19,20 and plated onto fibronectin-coated 6 well plate (Corning) with 1 × 10^6^ cells with Medium 199 (Gibco). Neonatal rat cardiomyocytes were incubated with CDC-CM, MSC-CM, or plain IMDM as the Control. After 3 days, cells were fixed with 4% PFA and apoptotic cells were detected by terminal deoxynucleotidyl transferase dUTP nick end labelling (TUNEL) assay using the In Situ Cell Death Detection Kit (Roche Diagnostics, Mannheim, Germany), according to the manufacturer’s instructions and DAPI for nuclei. Myocyte size quantification was performed by anti-α-SA antibody staining followed by cell area measurement using the NIH Image J software (Bethesda, MD, USA).

The pro-angiogenic effects of CDC-CM were studied by endothelial cell tube formation assay. Human umbilical vein endothelial cells (HUVECs; from ATCC) were seeded onto growth factor-reduced Matrigel™ (BD Biosciences) in 96-well plates at a density of 2 × 10^4^ cells per well. 100 μl of CDC-CM or MSC-CM were added into the wells. After 4 hrs, the wells were imaged with a Nikon (Chiyoda, Tokyo, Tokyo, Japan) white light microscope. The average tube length was then measured with NIH Image J Software.

### Animal procedures

All animal work is compliant with Institutional Animal Care and Usage Committee at North Carolina State University. We employed an acute doxorubicin-induced DCM model as previously described 21. Briefly, 6- to 8-week old female severe combined immunodeficiency mice (Charles River Laboratories, Wilmington, MA, USA) were given 10 mg/kg body weight intraperitoneally injected (bw) Doxorubicin to create acute DCM on Day 0. On Day 1, the mice were then randomized into the following two treatment groups (*n* = 9–11 mice per group): (*i*) Saline Control: Mice receiving 100 μl PBS injected through the tail vein; (*ii*) Cell Treatment: Mice receiving 100 μl of 1 × 10^6^ canine CDCs in 100 μl PBS injected through the tail vein. Blinded echocardiography was performed by a single observer, at day 0 and 7 after CDCs or PBS, for the measurement of cardiac function. Mice were anaesthetized with a 1.5% isofluorane-oxygen mixture and two-dimensional long axis images were record from the left caudal (apical) view, using a Philips CX30 ultrasound system couple with a L15 high-frequency probe. Two-dimensional guided M-mode images at *chordae tendineae* level were evaluated. M-mode measurements of left ventricle end-diastolic and end-systolic dimensions (LVEDD and LVESD, respectively) were performed by using the leading-edge method of the American Society of Echocardiograph 22. For estimation of each parameter, the average of three measurements from three different cycles in an image was obtained. Left ventricular end-diastolic and systolic volumes (LVEDV and LVESV, respectively) were calculated by the biplane method of disks (modified Simpson’s rule). Ejection fraction (EF) was determined by using (LVEDV – LVESV/LVEDV) × 100%, and fractional shortening (FS) was calculated from the M-mode echocardiography images as (LVEDD – LVESD/LVEDD) × 100%.

### Histology

All animals were killed 8 days after treatment. Mouse hearts were harvested and frozen in Optimal Cutting Temperature (OCT) compound (Tissue-Tek, Torrance, CA, USA). Cryo-sections (5 μm thick) were prepared. Masson’s trichrome staining was performed as per manufacturer’s instructions [HT15 Trichrome Staining (Masson) Kit; Sigma-Aldrich]. Fibrosis area was measured by NIH Image J as previously described 23. For immunofluorescence staining, mouse heart cryosections were fixed with 4% PFA, blocked/permeabilized with Protein Block Solution (DAKO) containing 1% saponin (Sigma-Aldrich), and then stained with anti-α-SA (Sigma-Aldrich) and anti-von Willebrand factor (Abcam) antibodies. FITC or Texas-Red secondary antibodies were obtained from Abcam and used in conjunction with these primary antibodies. Images were taken by a Zeiss (Jena, Germany) confocal microscopy system.

### Statistical analysis

Results are presented as mean ± SD unless specified otherwise. Comparisons between any two groups were performed with two-tailed unpaired Student’s *t*-test. Comparisons among more than two groups were performed with one-way anova followed by *post hoc* Bonferroni correction. Differences were considered statistically significant when *P* < 0.05.

## Results

### Generation of canine CDCs

**Figure 1:**
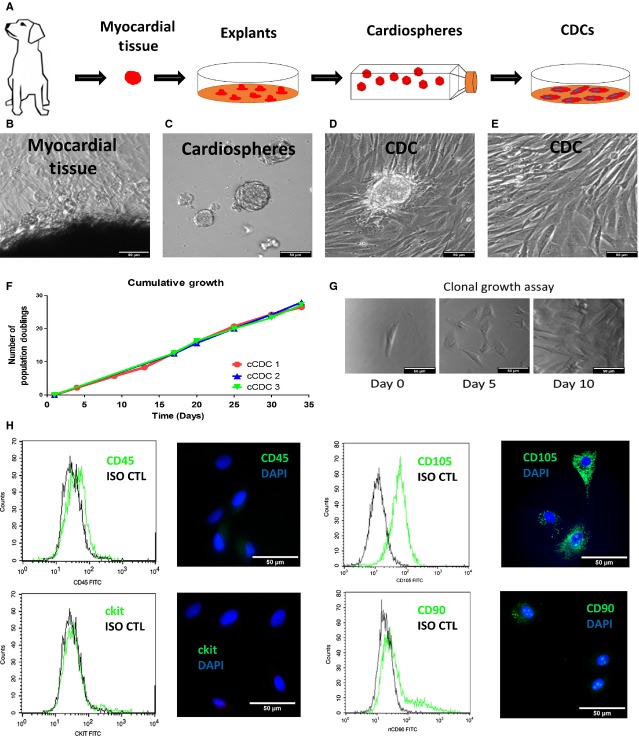
Derivation and culture of Canine CDCs. (A) Schematic diagram showing the derivation of canine CDC. (B) Outgrowth cells from plated myocardial tissues. (C) Cardiospheres forming in suspension culture. (D and E) Cardiosphere-derived cells (CDCs). (F) Cumulative growth analysis on population doublings of canine CDCs. (G) Clonal growth assay showing the progenies of a single canine CDCs at Day 1, 5 and 10. (H) Expressions of CD105, CD90, ckit, CD45 by flow cytometry and immunocytochemistry in canine. MSC data (Fig. S2); scale bars = 50 μm in all images.

Using a three-stage ‘adhesion-suspension-adhesion’ culture process (Fig.1A), we derived CDCs from dog myocardial tissues. Both phase-bright and stromal-like cells started to outgrow from the canine heart tissue explants in a week after plating onto fibronectin-coated surfaces. Those outgrowth cells become confluent in ∼2–3 weeks (Fig.1B). When seeded on low-attachment surfaces (to discourage cell attachment), the outgrowth cells spontaneously aggregate into three-dimensional canine cardiospheres (Fig.1C). To streamline the cardiosphere forming process, we used Ultra-Low attachment surface instead of the previously reported poly-d-lysine coating method. We demonstrated that cardiosphere formation and the potency of the resulted CDCs of the Ultra-Low method are indistinguishable from those of the poly-d-lysine method (Fig. S1). When replated onto a fibronectin-coated surface, the cardiospheres dissociated into single cells which we termed CDCs (Fig.1D and E). One biopsy-sized canine heart tissue can generate 50–200 millions of Passage 0 CDCs. When maintained and passaged in IMDM with 20% FBS, canine CDCs can further undergo 25–30 doublings in 35 days (Fig.1F). Clonal growth was observed in canine CDCs (Fig.1G). Flow Cytometry analysis and immunocytochemistry (Fig.1H) revealed that canine CDCs were highly positive for CD105 while being negative for haematopoietic marker CD45. Only a negligible fraction of canine CDCs express CD90 and ckit. As a positive control, canine MSCs uniformly express CD90 (Fig. S2).

### Differentiation potential of canine CDCs

**Figure 2:**
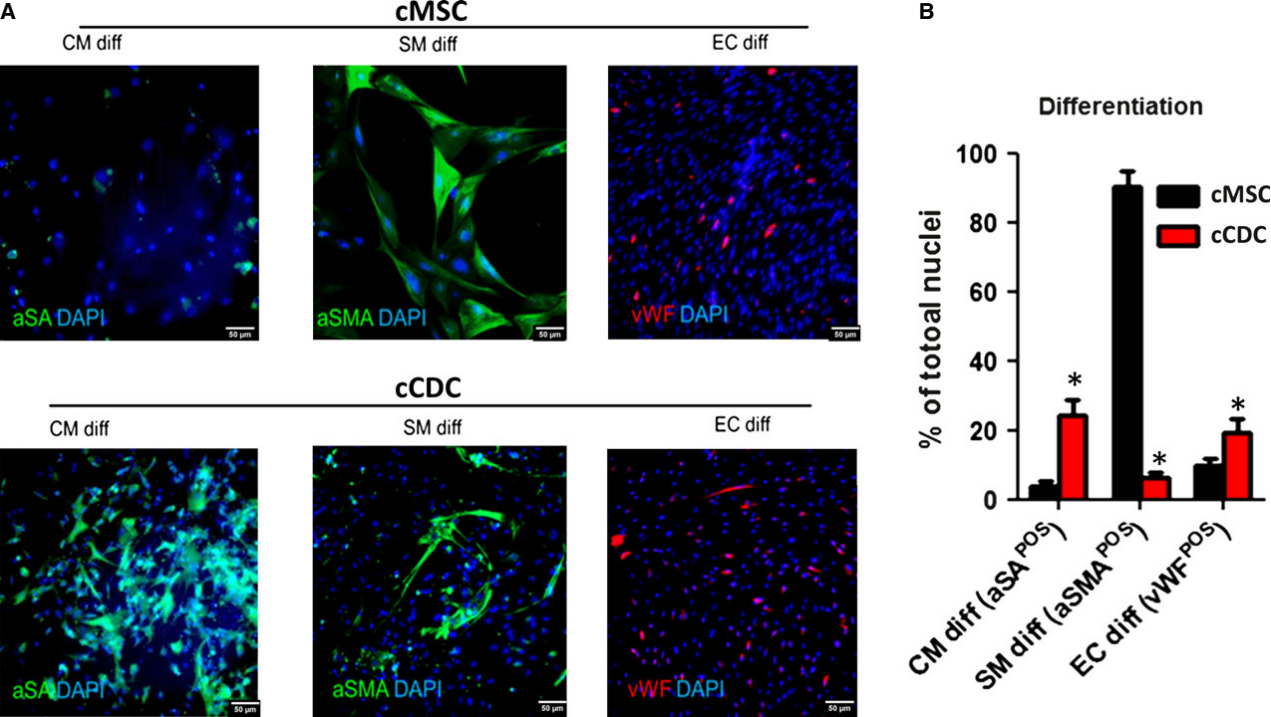
Cardiovascular differentiation of canine CDCs *in vitro*. (A) Representative fluorescent micrographs showing the expressions of aSA, aSMA and vWF in canine CDCs and MSCs r in three differentiation conditions: CM diff: cardiomyocyte differentiation; SM diff: smooth muscle differentiation; EC diff: endothelial cell differentiation. (B) Percentages of total cells that are positive for specific differentiation markers. * indicates *P* < 0.05 when compared to cMSC; scale bars = 50 μm.

After 12 days into differentiation, around 20% of canine C DCs started to express cardiomyocyte marker α-SA (Fig.2A and B) or cardiac troponin I (Fig. S3) while only ∼5% of canine MSCs expressed α-SA (Fig.2A and B). Instead, canine MSCs were potent in differentiation into smooth muscle cells: ∼90% of canine MSCs express alpha smooth muscle actin. The endothelial differentiation potentials of canine CDCs and MSCs were similar. These assays confirmed the cardiovascular differentiation potential of canine CDCs.

### Paracrine assays on canine CDCs

**Figure 3:**
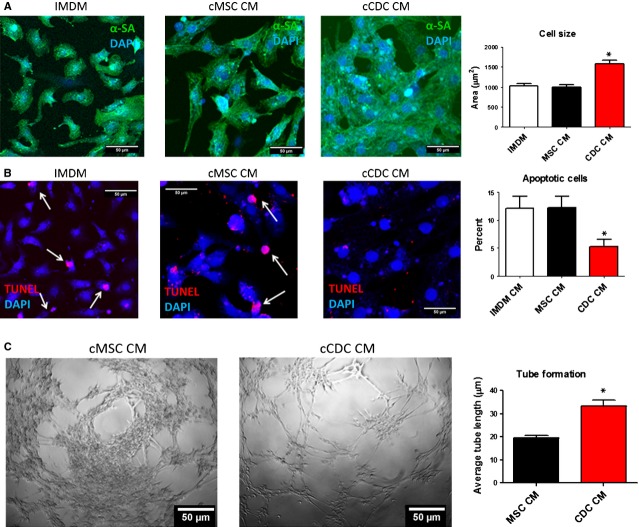
Paracrine assay. (A) Representative fluorescent micrographs showing neonatal rat cardiomyocytes (NRCM) grown in plain IMDM, condition media from canine MSC and condition media from canine CDC. Myocyte area were measured by NIH Image J software (*n* = 23). * indicates *P* < 0.05 when compared to MSC-CM or IMDM. (B) Representative fluorescent micrographs showing NRCMs grown in: plain media, condition media from canine MSC, condition media from canine CDC. Apoptotic cells were detected by TUNEL staining and quantified (*n* = 18). * indicates *P* < 0.05 when compared to cMSC CM or IMDM. (C) Measurement of average tube length in HUVEC cells cultured on Matrigel and incubated in conditioned media form canine CDCs or MSCs (*n* = 13). * indicates *P* < 0.05 when compared to cMSC CM; scale bars = 50 μm.

Incubation in CDC-CM increased the size/spreading of NRCMs (CDC-CM *versus* IMDM: 1586.2 ± 87.7 *versus* 1030.6 ± 52.5 μm^2^) (Fig.3A), while MSC-CM did not have any positive effects on cardiomyocyte size (997.1 ± 56.7 μm^2^). In addition, CDC-CM promoted the contractility of NRCMs as compared IMDM and MSC-CM (Videos S1–S3). In addition, CDC-CM protects cardiomyocytes from apoptosis: NRCMs incubated with IMDM and MSC-CM had a cell apoptosis rates (gauged by TUNEL staining) of 12.1 ± 2.1% and 12.3 ± 2.0%, respectively, while NRCMs incubated with CDC-CM had a markedly lower apoptosis rate of 5.3 ± 1.3% (Fig.3B). These compound data suggest canine CDCs are capable of promoting cardiomyocyte contraction and survival through paracrine mechanisms 24. Moreover, CDC-CM promotes tube formation of human umbilical vein endothelial cells on Matrigel™ (Fig.3C): average tube length in MSC-CM *versus* CDC-CM: 19.5 ± 1.2 *versus* 33.3 ± 2.6 μm, suggesting its pro-angiogenic role in cardiac regeneration.

### Canine CDC treatment improves cardiac function and reduces fibrosis

**Figure 4:**
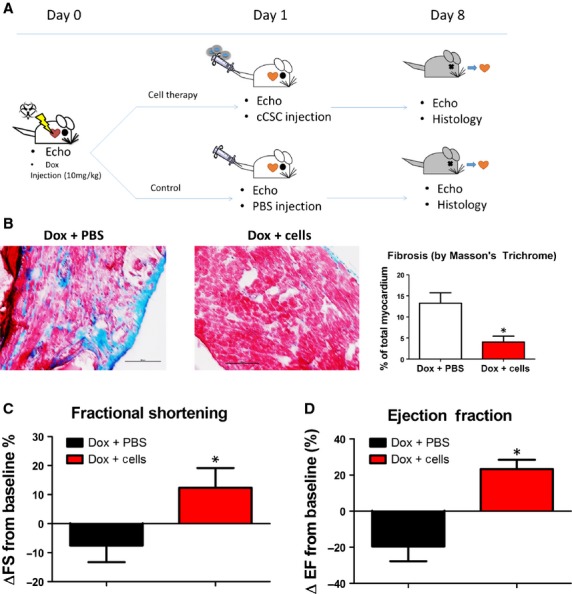
Cardiac function and fibrosis. (A) Schematic diagram showing the design of animal studies. (B) Representative Masson’s Trichrome staining images and quantification of fibrotic area (*n* = 3) Whole section view (Fig. S3A). (C and D) Change in ejection fraction (EF) and fractional shortening (FS) form baseline measurements (*n* = 9–11 animals per group); scale bars = 100 μm. *indicates *P* < 0.05 when compared to the Dox + saline treatment.

The animal study design is outline in (Fig.4A). Heart sections were stained with Masson’s Trichrome staining kit and analysed for fibrosis. Canine CDC injection significantly reduced fibrosis when compared to Control-treated hearts (Fig.4B, Fig. S3) (fibrosis area % of hearts in Control *versus* CDC therapy: 13.2 ± 1.4 *versus* 4.0 ± 0.8%). The *bona fide* therapeutic effects from stem cell therapy would be the improvement of heart pump functions. Echocardiography was performed at baseline and 7 days after cell or saline injection. Treatment effects (ΔEF% and ΔFS%) were calculated as the change in cardiac functions from the baseline. Saline injection had a negative treatment effects as EFs and FSs deteriorated over the 1 week time (Fig.4C and D, black bars). Cardiosphere-derived cell treatment (Fig.4C and D, red bars) protected cardiac functions from deteriorating because of the doxorubicin. These data suggest that canine CDC therapy mitigates fibrosis and pump function deterioration in DCM.

### Canine CDCs engraft in the mouse heart, promotion of angiogenesis and apoptosis reduction

**Figure 5:**
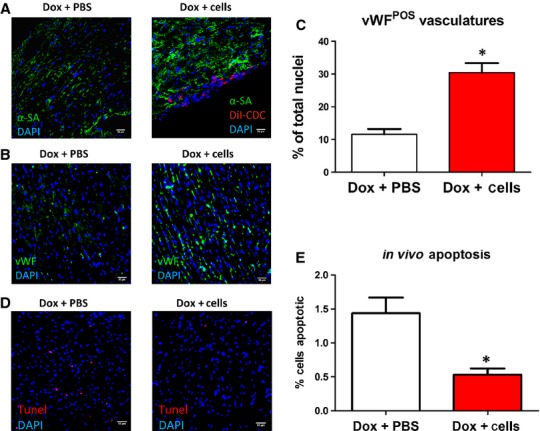
Canine CDC engraftment, pro-angiogenic effects, apoptosis measurement. (A) Representative micrographs showing the engraftment of DiI-positive CDCs in the DCM mouse heart. (B) Representative micrographs showing vWF-positive vasculatures. (C) Quantification of vWF-positive vasculatures (*n* = 9–14 animals per group). (D) Representative micrographs showing apoptosis. (E) Quantitation of cell apoptosis (*n* = 6). * indicates *P* < 0.05 when compared to Dox + Saline treatment; scale bars = 50 μm.

DiI-positive canine CDCs were detected in the mouse heart (Fig.5A). However, very few engrafted cells acquired mature cardiomyocyte or endothelial phenotypes. Mounting lines of evidence suggest that stem cell transplantation (including CDCs) exerts benefit through paracrine mechanisms, *i.e*. transplanted cells secrete factors to promote endogenous repair 24. Cardiosphere-derived cell therapy significantly increased vascular density in the DCM heart: vWF-positive vasculatures % of total nuclei in Control-treated hearts *versus* CDC-treated hearts: 11 ± 1.6 *versus* 30.4 ± 2.9% (Fig.5B and C). Cardiosphere-derived cell treatment reduced apoptosis in the post-MI hearts (TUNEL-positive cells in Control *versus* CDC therapy: 1.44 ± 0.23 *versus* 0.53 ± 0.09%) (Fig.5D and E).

## Discussion

Only a few families in the world are not affected by cardiovascular diseases. As a result of the magnitude-high prevalence-high incidence of cardiovascular disease in the world, stem cell therapy offers a promising option for therapeutic cardiac regeneration. The last decade witnessed a burst of cell therapy trials for ischaemic cardiomyopathy. For the last 6 years, our lab has been studying CDCs. Results from a recent clinical trial indicate that infusion of autologous CDCs in mild-to-moderate heart attack patients reduces scar and increases viable tissue. A Phase II clinical trial is ongoing to test the regenerative potential of allogeneic CDCs in patients with recent MI 25.

Veterinary patients such as domestic dogs suffer from heart diseases as well. For instance, it has recently been found that the overall prevalence of DCM in Doberman Pinschers is greater than 50% 18. The expected survival time after diagnosis is strikingly less than 2 months. The autosomal dominant inheritance pattern in Doberman Pinschers is similar to most forms of DCM in human’s heart 26,27. Once heart failure has occurred, treatment is only symptomatic and palliative. It is intriguing to wonder whether CDCs can be derived from donor dog hearts and used for therapeutic regeneration in dog patients with DCM. In addition, there is a growing interest from grant agencies such as National Institute of Health to use domestic dogs with naturally occurring heart diseases as model systems for cell-based cardiac regenerative therapies.

In this study, we demonstrate that canine CDCs can be derived from adult dog hearts and they are very phenotypically similar to human and rodent CDCs (Fig.1H). However, canine CDCs have low expressions in CD90 and ckit. Recent report from us and others has shown that ckit expression is irrelevant to the overall therapeutic benefit of CDCs and CD90 expression indeed undermines the regenerative potential of CDCs 18,28. Therefore, the low expressions in CD90 and ckit should not affect the therapeutic benefits of canine CDCs. Canine CDCs are cardiac stem cells and can express markers of cardiomyocytes, smooth muscle cells and endothelial cells *in vitro* (Fig.2). Consistent with our previous findings, the cardiac differentiation potential of canine CDCs is greater than canine MSCs 8.

Mounting lines of evidence indicate that CDCs exert their therapeutic benefit through paracrine mechanisms. We collected CM from canine CDCs and MSCs and study its effects on cardiomyocytes and endothelial cells. Compared to MSC-CM, CDC-CM is more potent in promoting myocyte contraction, inhibiting myocyte cell apoptosis and promoting endothelial cell tube formation on Matrigel (Fig.3). Additional staining using cardiac troponin I is also included in the supplemental data (Fig. S4).

We induced acute DCM in immunodeficiency mouse with injection of 10 mg/kg doxorubicin. This model has been reported to successfully induce DCM in less than 7 days 19. Injection of canine CDCs ameliorates ventricular dysfunction (Fig.4C) and reduces cardiac fibrosis (Fig.4B). Such observed therapeutic benefits are not accompanied by sizable cell engraftment: only a few of DiI-positive canine CDCs (Fig.5A) were detected in the mouse heart and even fewer differentiated into mature cardiac cells. The mechanisms underlying the therapeutic benefits of CDCs are not fully elucidated. It has been reported that CDCs are highly pro-angiogenic. Following this lead, we found that canine CDC treatment enhanced angiogenesis in the DCM heart (Fig.5B). Cardiosphere-derived cell treatment also reduced apoptosis when compared to no treatment (Fig.5D). The reduction in fibrosis may be a result of matrix metaloproteneases secreted by CDCs 29. It has also been reported that CDC therapy stimulates cardiomyocyte proliferation and recruitment of endogenous cardiac stem cells 9. Taking together, these mechanisms could explain the therapeutic benefits of CDCs in DCM.

In summary, we derived canine CDCs from adult dog hearts and showed their regenerative potential in a mouse model of induced DCM. Future studies will focus on testing canine CDCs in dogs with naturally occurring heart diseases such as DCM.

## Supporting Information

**Figure S1** Comparison of Poly-d-lysine and Ultra-Low methods for cardiosphere formation.

**Figure S2** Characterization of canine MSCs.

**Figure S3** Cardiac fibrosis.

**Figure S4** *In vitro* differentiation assay.

**Video S1** NRCMs cultured with CDC-CM.

**Video S2** NRCMs cultured with IMDM.

**Video S3** NRCMs cultured with MSC-CM.
