# Supplementary figures and images for: Cardiac regenerative potential of cardiosphere-derived cells from adult dog hearts
Source: J Cell Mol Med. 2015 Apr 9;19(8):1805–13. doi: 10.1111/jcmm.12585 (PMC4549031; doi:10.1111/jcmm.12585)

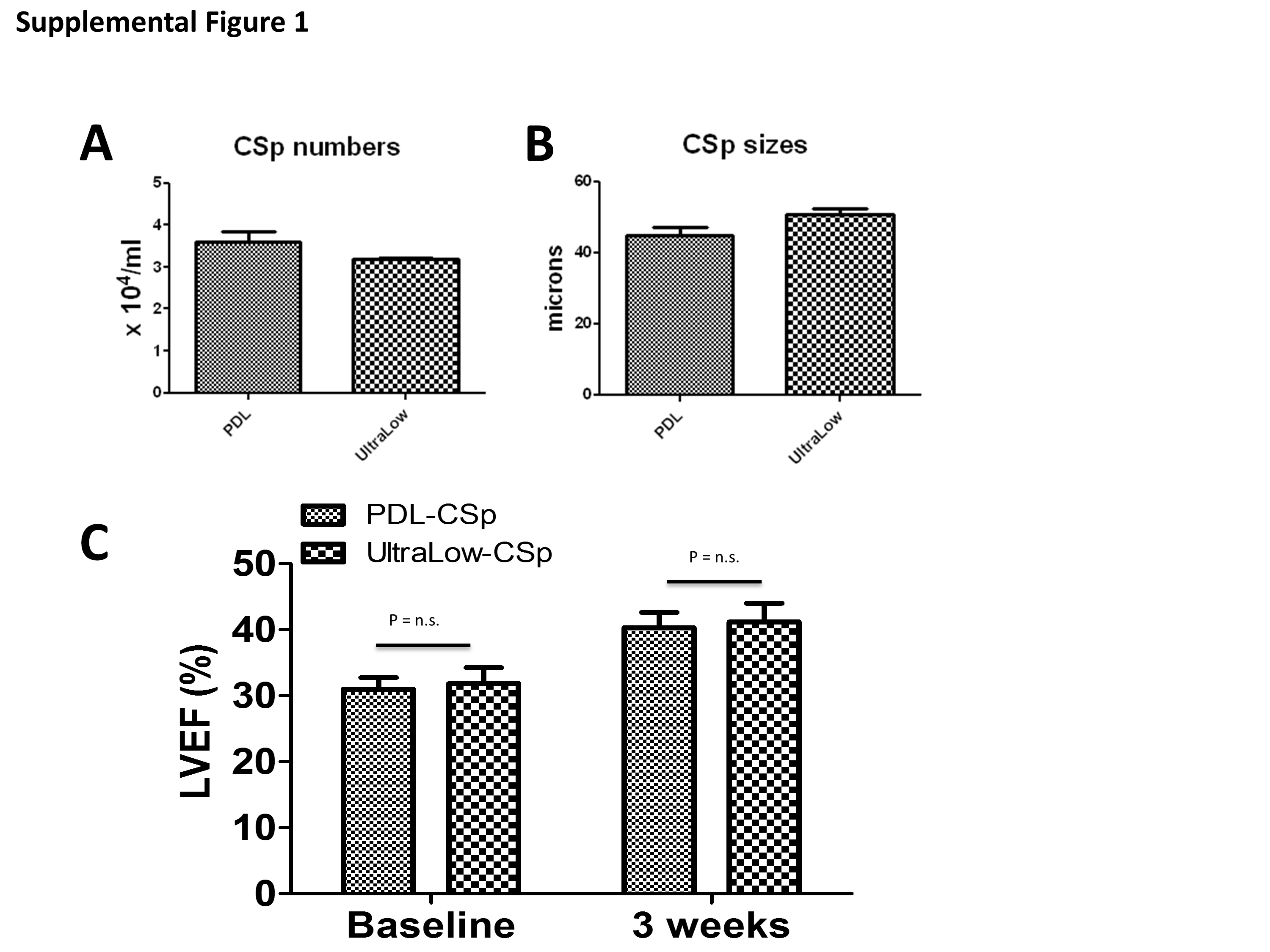

Supplement: Supplementary file 1 [file jcmm0019-1805-sd1.tif]

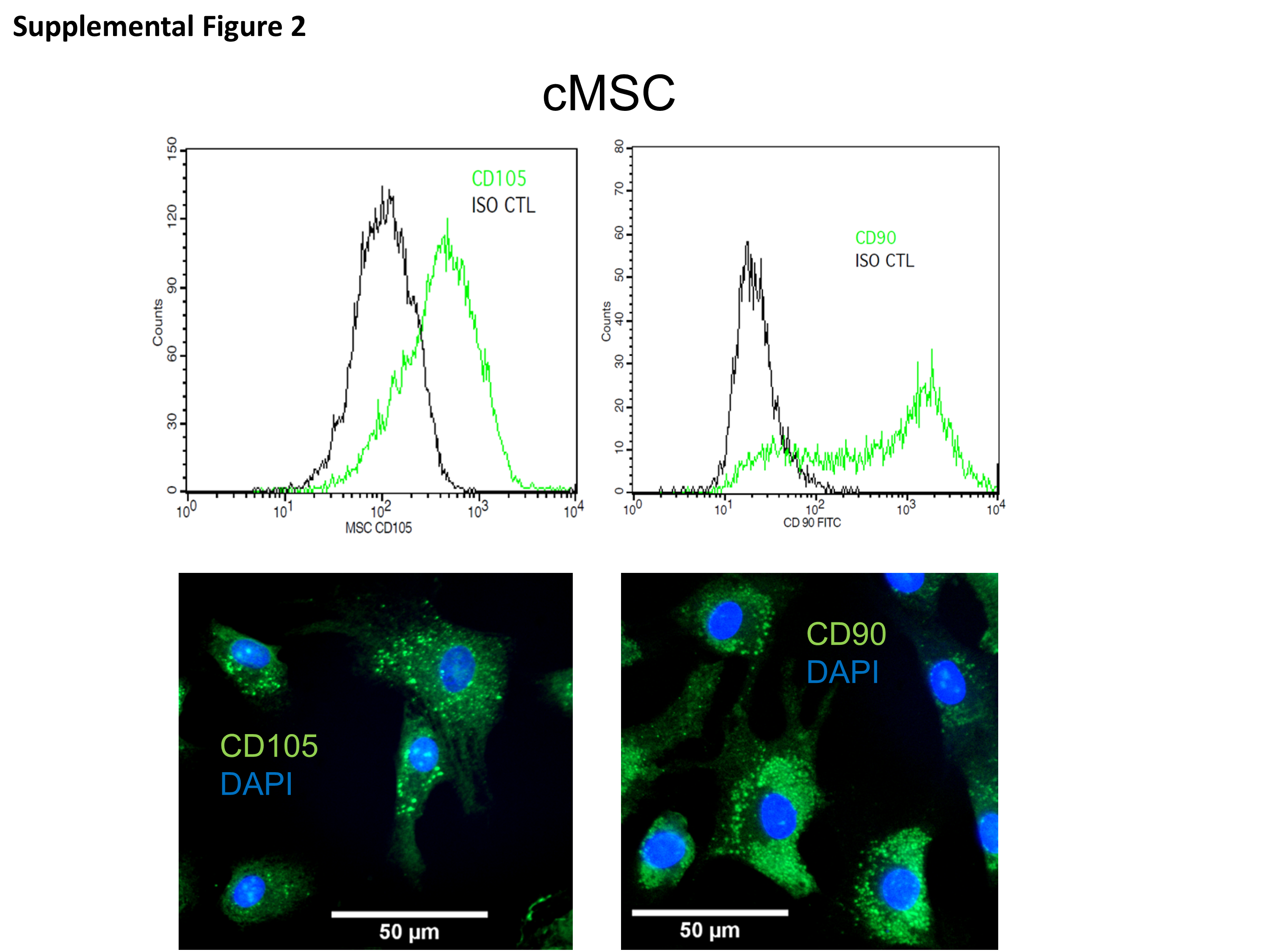

Supplement: Supplementary file 2 [file jcmm0019-1805-sd2.tif]

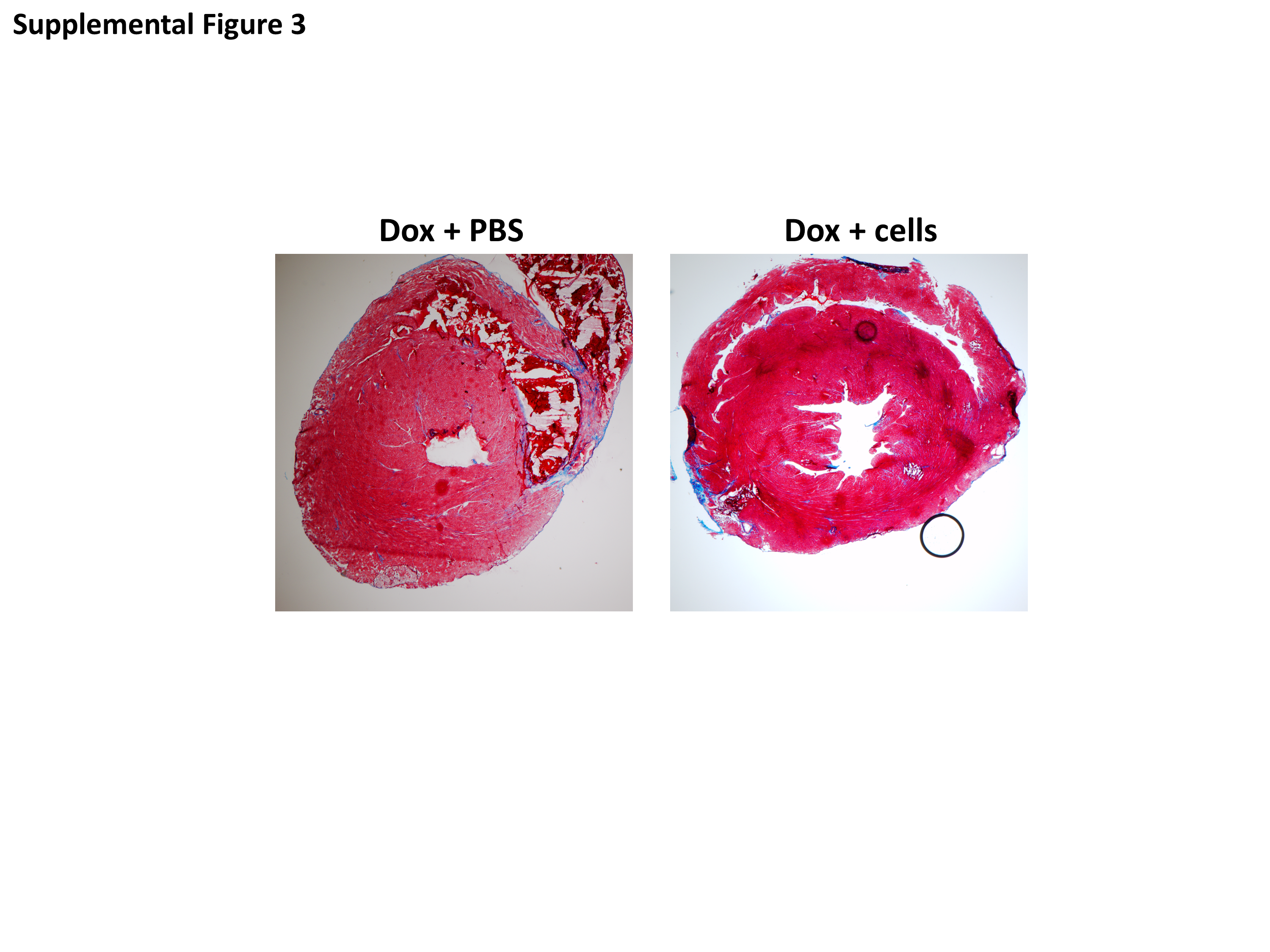

Supplement: Supplementary file 3 [file jcmm0019-1805-sd3.tif]

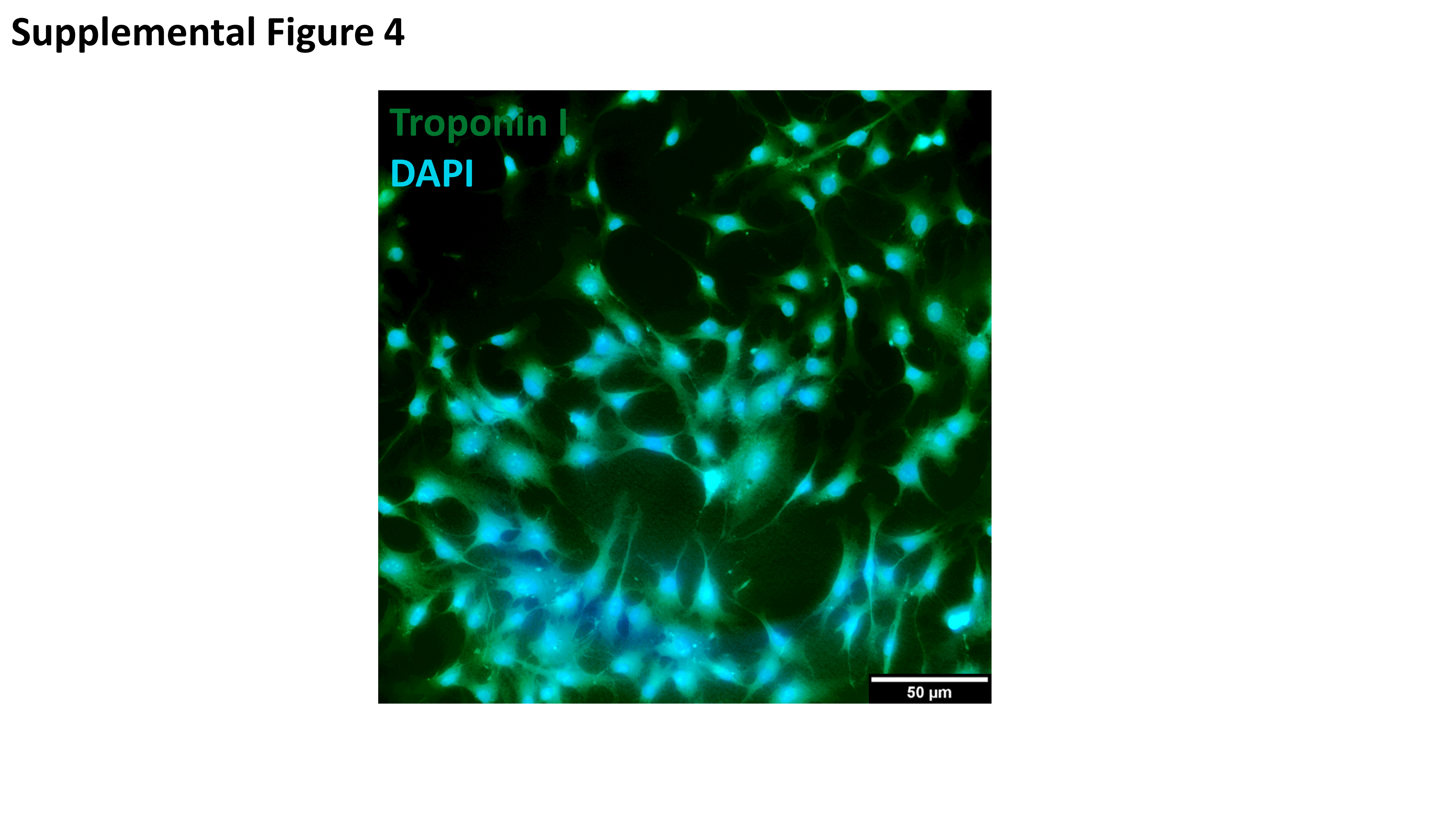

Supplement: Supplementary file 4 [file jcmm0019-1805-sd4.tif]
